# Comparing the Weighted Gain Score and a Rasch-Based Approach for Estimating Learning Outcomes in Medical Education: Quantitative Study

**DOI:** 10.2196/75516

**Published:** 2026-06-16

**Authors:** Rauf Aliyev, Joy Backhaus, Silke Hammer, Sarah König

**Affiliations:** 1 Institute of Medical Teaching and Medical Education Research, University Hospital Würzburg, Josef-Schneider-Str. 2/D6, Würzburg, 97080, Germany, +49 931 201 55210, +49 931 201 655213; 2 Institute of Diagnostic and Interventional Radiology, University Hospital Würzburg, Würzburg, Germany

**Keywords:** medical education, teaching quality, curriculum evaluation, learning gain, pretest-posttest design, Rasch model, Weighted Gain Score

## Abstract

**Background:**

Pretest-posttest designs are widely used to estimate learning gain in studies evaluating educational interventions in medical education. The Weighted Gain Score (WGS) was proposed to reduce bias associated with differences in baseline performance.

**Objective:**

This study evaluated the statistical and inferential properties of the WGS by comparing it to Rasch Learning Gain (RLG) across 3 datasets.

**Methods:**

The WGS implements a weighting coefficient that includes the parameter µ, which linearly rescales the difference between pretest and posttest percentage scores. We examined the effect of varying µ (30, 50, and 70) on learning gain calculations and compared the results with those obtained using RLG. The following three datasets were analyzed: (1) a small illustrative dataset demonstrating the mathematical behavior of the WGS, (2) an empirical dataset from a previous educational evaluation study, and (3) a randomly generated binomial dataset designed to examine the metric under larger sample conditions.

**Results:**

Changing the parameter µ in the WGS affected the magnitude of the calculated learning gains: lower µ-values produced larger gain estimates, whereas higher µ-values produced smaller estimates. Despite these differences in scale, the WGS and RLG correlated strongly in both the empirical dataset (*r*=0.93; *P*<.001) and the simulated dataset (*r*=0.92; *P*<.001); variation in µ did not alter the inferential results. Both methods identified the same interaction effect in the empirical dataset.

**Conclusions:**

The WGS produced results highly consistent with those of RLG while requiring substantially lower computational complexity. The metric can be applied to both small and large datasets and allows µ to function as an adjustment coefficient for calibrating learning gain estimates across cohorts without altering inferential conclusions.

## Introduction

Teaching quality in medical education is a complex construct encompassing curriculum design, instructional methods, teaching expertise, learner engagement, and assessment practices [1-4]. High-quality teaching in this context contributes to the development of competent physicians and thereby influences the quality of patient care [5,6].

Among the various aspects of teaching quality in medical education, student learning outcomes represent one measurable indicator frequently used in program evaluation and educational research [7-11]. However, interpreting learning outcomes as indicators of teaching effectiveness requires caution, as they are influenced by multiple factors beyond instructional quality. These include student motivation, prior knowledge, learning strategies, teacher enthusiasm, and learning activities occurring outside the formal curriculum [12,13]. To account for these influences, educational research often focuses not only on absolute performance but also on changes in performance over time. The concept of learning gain represents a widely used approach to capturing students’ learning progress. In educational research, learning gain is commonly operationalized by assessing students before (pretest) and after (posttest) an educational intervention. The difference between pretest and posttest scores is then interpreted as an indicator of learning gain attributable, at least in part, to the educational intervention [14-16]. However, calculating learning gain is not trivial, as simple difference scores may lead to biased estimates depending on students’ baseline knowledge. One simple approach is raw gain, which is calculated as the arithmetic difference between posttest and pretest scores. However, raw gain scores exhibit a negative correlation with baseline performance (ie, pretest scores) and are also affected by ceiling effects, meaning that students with lower pretest scores may appear to exhibit larger gains simply because they have more room for improvement [17-19].

To address these limitations, several modified gain metrics have been proposed. One widely used approach is the normalized gain introduced by Hake [20], which expresses the observed pretest-posttest gain relative to the maximum possible gain. Although this metric has been applied extensively in educational research [21,22], it also has important methodological limitations. It remains dependent on baseline performance, may inflate gains for students with high pretest scores, and behaves inconsistently when posttest scores fall below pretest scores or when pretest scores approach the maximum value [16,23].

Taken together, existing gain metrics may distort estimates of learning gain, particularly in cohorts with heterogeneous baseline knowledge. Many of these metrics either remain strongly dependent on baseline performance or require complex psychometric modeling. This highlights the need for approaches that provide statistically robust yet practically applicable estimates of learning gain in educational evaluation.

A recently proposed metric developed by our workgroup, the “Weighted Gain Score” (WGS), aims to address these limitations by applying a weighting coefficient that adjusts gain calculations according to students’ baseline performance [16]. However, the statistical and inferential properties of this metric have not yet been systematically investigated. To address this gap, we evaluated the WGS by comparing it with Rasch Learning Gain (RLG), a Rasch model–based approach for estimating learning gain that served as the benchmark in our study [24]. Specifically, we addressed the following research questions:

- Does the WGS produce inferential results comparable to those produced by RLG?
- Can the parameter µ in the WGS be adjusted for different cohorts to calibrate learning gain calculations without altering inferential conclusions?

Through this analysis, we aimed to clarify the statistical behavior of the WGS and explore its potential applicability for the evaluation of educational interventions.

## Methods

### Metric WGS

The mathematical foundation of the WGS lies in the use of the weighting coefficient “pre/µ,” which linearly transforms the difference between pretest and posttest percentage scores (denoted as “pre” and “post” in equation 1), thereby adjusting for pretest variability [16]. Formally, the WGS is defined as:


(1) WGS=(post−pre)×(pre/μ)

To illustrate the computation, consider a hypothetical student with a pretest score of 40% and a posttest score of 70%.

For µ=50, the WGS is calculated as: WGS = (70 – 40) × (40/50) = 30 × 0.8 = 24.

If µ is increased to 70, the same performance yields: WGS = (70 – 40) × (40/70) = 30 × 0.57 = 17.14.

This example illustrates that increasing µ reduces the magnitude of the calculated gain while preserving the relative ordering of observations. When posttest scores fall below pretest scores, the WGS assumes negative values, indicating a decrease in performance.

Originally, the parameter µ used in the weighting coefficient was defined as the average pretest score of a cohort. It was constrained to integer values between 1 and 100, consistent with the percentage format of “pre” and “post.” In the original formulation, its value was set at 50 as a default reference value [16]. In this study, µ is interpreted as an adjustment coefficient that functions as a scaling parameter for learning gain calculations. Changing its value proportionally rescales the calculated gain scores: higher values of µ lead to smaller gain estimates, whereas lower values produce larger gain estimates. Importantly, this modification represents a linear transformation of the calculated values and therefore does not alter the underlying statistical relationships among observations.

To examine the influence of this parameter on the stability of the WGS, we tested 3 calibration levels in our datasets: µ=30, µ=50, and µ=70. These values represent 3 nonextreme points within the possible range of 1 to 100, allowing us to evaluate the behavior of the WGS across low, moderate, and high scaling conditions.

### Rasch Model and RLG

The Rasch model is a fundamental concept in modern psychometric measurement. The probability that a student answers a specific item correctly depends on 2 key factors: the student’s ability and the difficulty of the item. In the Rasch framework, a student’s latent ability is denoted by θ, whereas item difficulty is represented by β. When a student’s ability exceeds the difficulty of an item, the probability of answering correctly increases, and vice versa [25]. Because the Rasch model allows the estimation of individual students’ abilities independently of the specific test items used, it is widely applied in educational measurement and medical education research [26]. With this in mind, we selected RLG as a reference method for evaluating the WGS.

We applied the dichotomous 1-parameter logistic Rasch model. Item parameters were estimated using conditional maximum likelihood estimation. On the basis of the fitted model, person abilities were subsequently calculated using maximum likelihood estimation separately for the pretest (θ_pre_) and posttest (θ_post_) data [27].

As indicated in equation 2, RLG was defined as the difference between the estimated posttest and pretest abilities. This difference represents the change in latent ability on the Rasch measurement scale and serves as an estimate of individual learning gain across the instructional intervention [24,28].


(2) $$RLG=(\theta_{post}-\theta_{pre})$$

To ensure the validity of Rasch-based ability estimates, we examined global model fit indicators. Item infit and outfit statistics ranged between 0.7 and 1.3, which is generally considered acceptable for the Rasch model. In addition, person reliability exceeded 0.8, and separation indices were >2, indicating satisfactory measurement precision.

### Datasets

Three datasets were used to examine the behavior of the WGS under different analytical conditions:

1. The illustrative dataset (n=10): a small artificial dataset designed to illustrate the mathematical behavior of the parameter µ within the WGS metric
2. The empirical dataset (n=170): a dataset consisting of real-world data derived from a previously published educational evaluation study [29], used to examine the behavior of the WGS under authentic educational conditions and to perform inferential statistical analyses
3. The simulated dataset (n=1000): a randomly generated binomial dataset designed to mirror the structure of the empirical dataset while providing a larger sample size, allowing the behavior of the parameter µ to be examined independently of the empirical data

### The Illustrative Dataset

Following the design of the simulated dataset in our previous study [16], we created an artificial dataset by combining different pretest scores with varying levels of raw gain in test performance, defined as the absolute difference between posttest and pretest scores. Pretest scores ranged from 1 to 10 points, and the gain in performance was simulated by increasing test scores by 1 to 4 points. To avoid potential ceiling effects, the analysis included only combinations in which the sum of pretest scores and the simulated gains did not exceed the maximum of 10 points. The sample size of the illustrative dataset was set at 10. RLG was not applicable here, as Rasch model–based estimation requires larger sample sizes to obtain stable parameter estimates [30].

### The Empirical Dataset

The empirical dataset originated from a prospective educational study conducted at the University Medical Center Göttingen in Göttingen, Germany [29]. The study compared the learning gain of students attending a traditional lecture on goiter with that of students using a corresponding video podcast (vodcast) within the teaching module “Operative Medicine.” The study was conducted over 2 consecutive semesters using a pretest-posttest design based on 9 multiple-choice test items. A total of 170 students participated. Students were additionally surveyed regarding their learning dispositions, which resulted in the classification of participants into 2 groups: “traditional learners” and “digital natives.” A total of 35 students (20.59%) could not be clearly assigned to either group and were therefore excluded from group-based analyses. Consequently, 135 (79.41%) students were included in the 2-way ANOVA examining the interaction between teaching format and learning disposition (Table 1).

**Table 1.** Distribution of students according to teaching format and learning disposition in the empirical and simulated datasets.

Datasets and teaching formats	Traditional learners, n (%)	Digital natives, n (%)
Empirical dataset (N=135)		
Lecture	38 (28.15)	34 (25.19)
Vodcast	28 (20.74)	35 (25.93)
Simulated dataset (N=1000)		
Lecture	259 (25.9)	210 (21)
Vodcast	250 (25)	281 (28.1)

### The Simulated Dataset

The simulated dataset was generated using a random binomial distribution, assuming a 50% probability of correctly answering a hypothetical examination item. This probability was applied to 9 multiple-choice items in both the pretest and the posttest scores, reflecting the structure of the “empirical dataset.” Apart from the larger sample size, the primary difference between the empirical and simulated datasets was the random allocation of group variables. Two variables were simulated: teaching format and learning disposition. Both variables were coded dichotomously. For consistency in labeling, the simulated variables were named analogously to those in the empirical dataset, although they represent random group assignments rather than actual instructional formats or learning characteristics. Each simulated student had a 50% probability of being assigned to each category (Table 1). The sample size for the simulated dataset was 1000.

### Statistical Analysis

All simulations and statistical analyses were conducted using the R software suite (version 4.1.2; R Foundation for Statistical Computing) [31]. Rasch modeling was performed using the *eRm* package [32].

To examine the relationship between the 2 learning gain metrics, Pearson correlation coefficients were calculated between the WGS and RLG scores.

To investigate potential interaction effects between teaching format and learning disposition, we conducted a 2-way ANOVA. Post hoc comparisons were performed using Bonferroni-adjusted contrasts. Effect sizes were reported as partial η², and 95% CIs were calculated where appropriate.

Normality of the dependent variables was assessed using the Shapiro-Wilk test and visual inspection of Q-Q plots. Minor deviations from normality were observed, which are common in bounded percentage scores (0% to 100%) frequently used in educational assessments. Given the present sample sizes and the absence of influential outliers, ANOVA was considered sufficiently robust to moderate violations of the normality assumption.

Homogeneity of variances across groups was evaluated using the Levene test and the Brown-Forsythe test, both of which indicated no statistically significant differences in variance between groups. All statistical tests were 2-sided, and a significance level of *P*<.05 was applied.

### Ethical Considerations

The empirical data analyzed within this work were reviewed and judged by the local institutional review and ethics board (Medical Ethics Committee, University Medical Center Göttingen) as not representing medical or epidemiological research on human participants and, therefore, were assessed using a simplified assessment protocol. The project was approved without any reservation under proposal number 1-11-14.

## Results

### Effect of the Parameter µ on WGS Learning Gain Estimates

**Figure 1.**
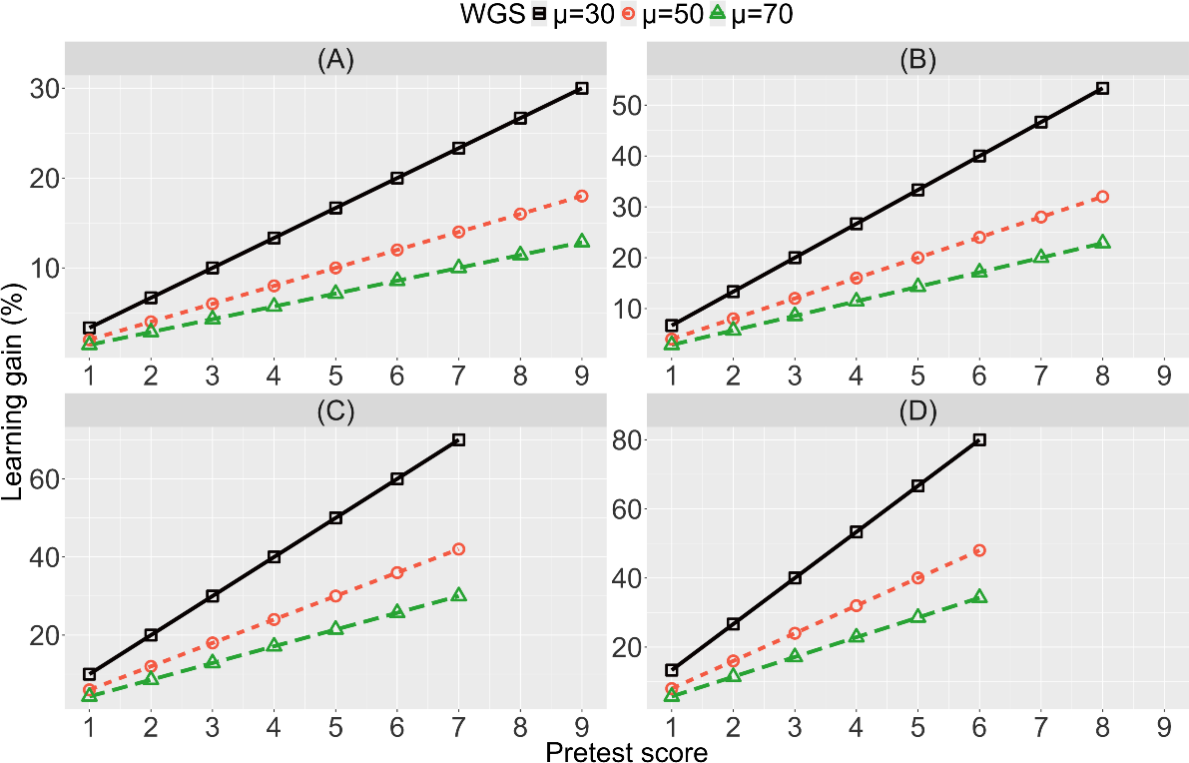
Effect of varying µ (30, 50, and 70) on Weighted Gain Score (WGS) learning gain estimates in the illustrative dataset. (A) Gain of 1 point, (B) gain of 2 points, (C) gain of 3 points, and (D) gain of 4 points.

The illustrative dataset demonstrates the mathematical effect of varying µ (30, 50, and 70) on the WGS. Changes in µ systematically altered the slope of the WGS learning gain plots (Figure 1). Each subplot represents a different raw gain scenario, ranging from 1 to 4 points. As the µ-value increased, the slope of the learning gain curve decreased, resulting in smaller WGS values for the same pretest score. For example, with a gain of 1 point and a pretest score of 6, the WGS was approximately 20% for µ=30 and decreased to <10% for µ=70. This pattern remained consistent across all 4 gain scenarios, illustrating that increasing µ reduces the magnitude of the calculated learning gain while preserving the relative ordering of observations.

### Correlation Analysis Between WGS and RLG

**Figure 2.**
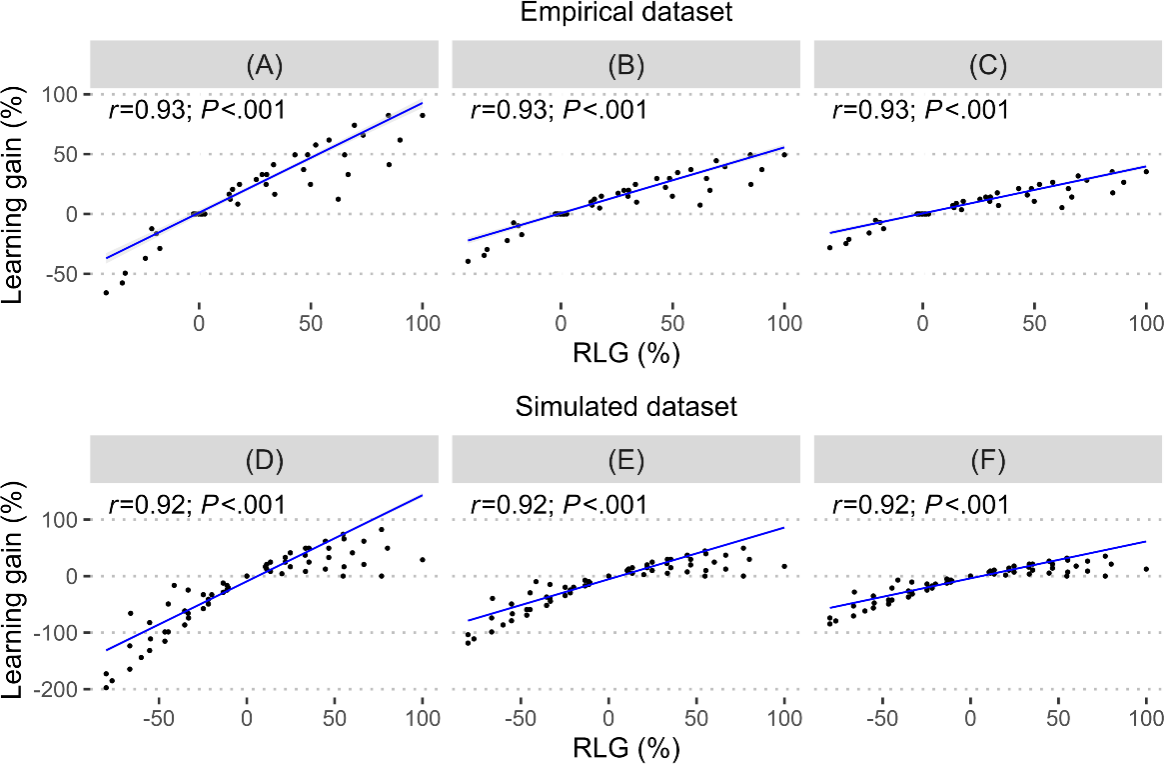
Correlation between Weighted Gain Score (WGS) and Rasch Learning Gain (RLG) in the empirical and simulated datasets. (A) Empirical dataset with µ=30, (B) empirical dataset with µ=50, (C) empirical dataset with µ=70, (D) simulated dataset with µ=30, (E) simulated dataset with µ=50, and (F) simulated dataset with µ=70.

The WGS demonstrated a strong positive correlation with RLG across all tested µ-values (Figure 2). In the empirical dataset, the Pearson correlation coefficient was consistently high (*r*=0.93; *P*<.001). A similarly strong relationship was observed in the simulated dataset (*r*=0.92; *P*<.001). The correlation coefficients remained identical across the tested µ-values (30, 50, and 70) in both datasets.

### Analysis of Interaction Effects Using WGS and RLG

**Figure 3.**
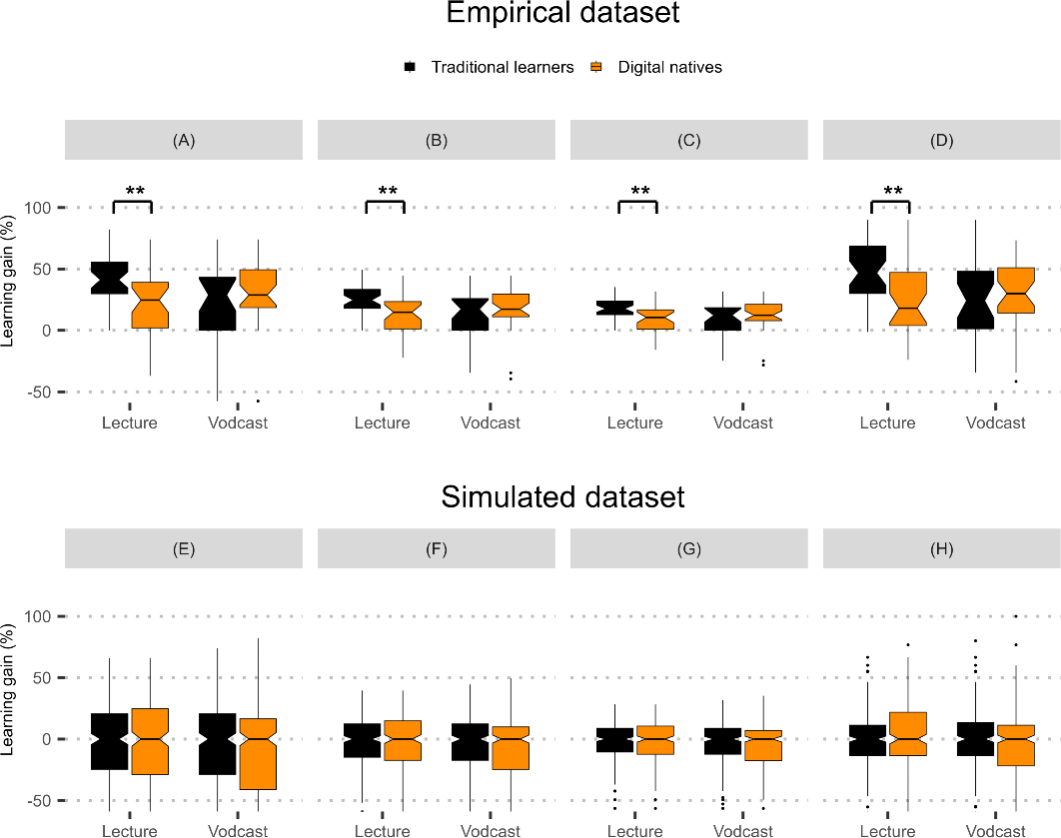
Learning gain estimates calculated using Weighted Gain Score (WGS) and Rasch Learning Gain (RLG), depicting the interaction between teaching format and learning disposition in the empirical and simulated datasets. (A) Empirical dataset with WGS (µ=30), (B) empirical dataset with WGS (µ=50), (C) empirical dataset with WGS (µ=70), (D) empirical dataset with RLG, (E) simulated dataset with WGS (µ=30), (F) simulated dataset with WGS (µ=50), (G) simulated dataset with WGS (µ=70), and (H) simulated dataset with RLG. **Indicates statistical significance at *P*=.01.

All 3 calibrations of the WGS (µ=30, µ=50, and µ=70) detected a significant interaction effect between teaching format and learning disposition in the empirical dataset (Figure 3). Traditional learners displayed higher learning gains in the lecture format than digital natives (*F*_1,131_=6.51; *P*=.01; partial η²=0.05). For µ=50, the mean difference was −11.64 (95% Bonferroni-adjusted CI −21.46 to −1.83; *P*=.01). Corresponding estimates were −19.41 for µ=30 (95% Bonferroni-adjusted CI −35.80 to −3.04; *P*=.01) and −8.32 for µ=70 (95% Bonferroni-adjusted CI −15.33 to −1.31; *P*=.01).

RLG also detected this interaction effect (*F*_1,131_=6.75; *P*=.01; partial η²=0.05) with a mean difference of −19.91 (95% Bonferroni-adjusted CI −36.80 to −3.05; *P*=.01), confirming the interaction pattern observed in the original study from which our empirical dataset was derived [16,29].

In the simulated dataset, no significant interaction between teaching format and learning disposition was observed when learning gains were calculated using the WGS, regardless of the µ-value applied (*F*_1,996_=0.39; *P*=.53; partial η²<0.001; Figure 3). Similarly, RLG did not reveal any significant difference in performance between the groups (*F*_1,996_=1.10; *P*=.29; partial η²=0.001). Because teaching format and learning disposition were randomly assigned in the simulated dataset, we did not necessarily expect any interaction effect.

## Discussion

### Inferential Behavior of WGS Compared With RLG

A robust method for calculating learning gain is essential for capturing students’ learning progress following an educational intervention and for providing interpretable indicators of educational effectiveness. Such a method should be statistically sound, transparent, and practically applicable within evaluation processes.

This study evaluated the statistical behavior of the WGS, a method designed to estimate learning gain in a way that is both methodologically robust and straightforward to implement. The first research question examined whether the WGS yields inferential results comparable to those obtained with RLG. Our findings demonstrated a strong inferential correspondence between the 2 methods. The WGS produced learning gain estimates that correlated highly with those derived from RLG, while also identifying the same interaction effect in the empirical dataset as the Rasch model–based approach. Importantly, these inferential conclusions remained stable across all tested µ-values (30, 50, and 70). The identical correlation coefficients between the WGS and RLG and the unchanged ANOVA results indicate that modifying the parameter µ linearly rescales learning gain estimates. Consequently, varying µ changes the magnitude of WGS values but does not affect statistical inference.

### Robustness of WGS Under Nonnormally Distributed Data

Neither the empirical nor the simulated dataset fully satisfied the assumption of normality, although no substantial skewness was observed. In medical education research, deviations from normality are common, particularly in pretest-posttest designs [17,33]. A ceiling effect occurs when pretest scores approach the maximum possible value, limiting the measurable improvement, whereas a floor effect arises when pretest performance is concentrated near the minimum score in a difficult test. Very easy items tend to produce ceiling effects, whereas very difficult items may lead to floor effects. Despite deviations from normality, the WGS demonstrated stable inferential behavior across the empirical and simulated datasets, suggesting a degree of robustness. This finding is consistent with previous research indicating that parametric methods such as ANOVA and correlation analyses are generally robust to moderate violations of normality, particularly in samples of the size examined in this study [34-36]. Nevertheless, future research is needed to examine the behavior of the WGS across a broader range of distributional scenarios to better establish its reliability.

### Applicability of WGS in Small Samples

The illustrative dataset demonstrates that the WGS yields interpretable results even with very small sample sizes. In contrast, Rasch model–based approaches typically require substantially larger samples to ensure stable estimation of item parameters and person abilities [24,26]. This distinction is particularly relevant in educational settings with small cohorts, such as specialized teaching modules, pilot courses, or resource-intensive instructional interventions. In such contexts, the WGS may represent a practical alternative method for estimating learning gain because it does not rely on complex parameter estimation.

More broadly, transparent feedback on learning outcomes supports the continuous development of teaching practices, as evidence suggests that feedback on educational performance encourages educators to engage in reflective improvement of their teaching [37-39].

### The Role of µ as an Adjustment Coefficient

The second research question examined whether the parameter µ in the WGS can be adjusted across different cohorts to calibrate learning gain calculations without altering inferential outcomes. In the original study introducing the WGS, µ was defined as the average pretest score of a cohort. Our findings suggest that the role of µ can be understood more broadly. Rather than representing solely the cohort mean, µ functions as a scaling parameter that allows calibration of the learning gain metric. To reflect this role more accurately, we interpret µ in this study as an adjustment coefficient that can be modified depending on the analytical purpose of the evaluation. On the basis of the results of this study, 3 conceptual adjustment strategies can be distinguished: absolute adjustment, relative adjustment, and routine evaluation. A decision framework for selecting µ is provided in Multimedia Appendix 1.

### Absolute Adjustment: Monitoring of Cohort Learning

Absolute adjustment refers to the use of a fixed µ-value to estimate learning gain within a stable scaling framework. When µ remains constant, differences in learning gain across courses, time points within the curriculum, or different cohorts can be interpreted without recalibration of the metric, thereby ensuring cross-cohort comparability. This approach supports standardized monitoring of educational outcomes, for example, when evaluating curricular developments over time or comparing modules within a program. Observed differences in learning gain may arise from multiple factors, including instructional design, assessment characteristics, or cohort composition. Maintaining a fixed µ ensures that such differences remain visible and can be attributed to substantive factors.

### Relative Adjustment: Evaluation of Teaching Interventions

Relative adjustment enables comparison of teaching interventions across cohorts with heterogeneous characteristics. In educational practice, cohorts often differ in characteristics such as demographics, motivation, workload, or external contextual influences [13,40]. When learning gain is used to compare instructional formats, such heterogeneity may affect the interpretation of outcomes. Under a relative adjustment strategy, a µ-value may be calibrated separately for each cohort, allowing the scaling of learning gain calculations to reflect cohort-specific baseline conditions. Although this approach does not eliminate potential confounding factors, it may reduce systematic bias associated with heterogeneous starting conditions. This strategy is particularly useful when learning gain is evaluated without strict requirements for cross-cohort comparability, but with a focus on fair comparison of teaching interventions within specific cohorts or instructional contexts.

### Routine Evaluation: Selecting µ in Practice

In routine applications, when learning gain is estimated without strict requirements for cross-cohort comparability or cohort-specific calibration, µ may be selected pragmatically based on the cohort’s mean pretest performance. For example, cohorts with mean pretest scores approximately 50% of the maximum achievable score may be assigned µ=50, whereas cohorts with substantially higher or lower baseline knowledge may be assigned correspondingly higher (eg, ≥70) or lower (eg, ≤30) µ-values. This pragmatic approach enables straightforward estimation of learning gain while preserving a transparent and easily interpretable scaling of the WGS metric.

### Limitations

One limitation of the WGS arises when a student obtains a pretest score of zero, which results in a calculated learning gain of zero regardless of posttest performance. In practice, such cases are unlikely in multiple-choice assessments because guessing and prior knowledge increase the probability of obtaining at least 1 correct response [41]. In the empirical dataset, no student recorded a pretest score of zero, and in the simulated dataset, a negligible number (3 out of 1000 students) achieved zero points on the pretest score. One possible strategy is to exclude such observations from the analysis. However, this may reduce statistical power and introduce bias if students with low baseline scores are systematically underrepresented. Alternatively, a small positive offset (pseudocount) could be added to avoid undefined computations, analogous to continuity corrections used in categorical data analysis [42,43]. The implications of such adjustments should be examined in future methodological studies, for example, through sensitivity analyses comparing different handling strategies for zero-baseline observations [44].

A further limitation concerns the sample size of the empirical dataset (n=170). Although cohort sizes of this magnitude are common in single-semester cohorts at German medical faculties, they are slightly below commonly cited recommendations for stable Rasch parameter estimation, which often suggest sample sizes of approximately 150 to 200 participants or more [45]. Nevertheless, global model fit indicators in the empirical dataset (infit and outfit statistics, person reliability, and separation indices) were within acceptable ranges, supporting the interpretability of the RLG-based estimates despite the moderate sample size.

Another limitation relates to the test length used in the simulated dataset, which consisted of 9 multiple-choice items to mirror the empirical dataset. Because measurement reliability generally increases with test length [46-50], the limited number of items may reduce measurement precision and restrict the generalizability of the findings. Therefore, future research should examine the performance of the WGS in assessments with larger item sets that more closely reflect the scope of medical examinations.

Finally, both datasets exhibited deviations from normality**,** although homogeneity of variances across groups was supported by the Levene and Brown-Forsythe tests, and no influential outliers were observed. Previous methodological research indicates that ANOVA and Pearson correlation are generally robust to moderate violations of normality, particularly in samples of the present size [34-36]. Therefore, we consider the impact of nonnormality on the inferential conclusions to be limited.

### Conclusions and Future Research

This study evaluated the WGS as a method for estimating learning gain in pretest-posttest educational designs. Our findings indicate that the WGS provides robust and easily interpretable estimates while remaining computationally simple. Rather than replacing established psychometric models, the WGS may complement existing approaches, particularly in routine educational evaluations.

Future research should further develop the WGS as a broadly applicable evaluation instrument. In particular, establishing a methodologically sound calibration framework for µ will be essential, including empirically grounded decision models that guide µ-selection according to the evaluation purpose, such as cohort monitoring or comparative evaluation of teaching interventions. In addition, integrating the WGS into structured program evaluations, including longitudinal monitoring across courses, will be important for assessing its generalizability across educational contexts.

Future work may also explore the integration of the WGS within Bayesian test-theoretical frameworks [51]. By incorporating prior information and updating gain estimates as new data become available, Bayesian approaches could further improve the precision and contextual sensitivity of WGS-based learning gain estimates. Further studies should also examine the behavior of the WGS under different distributional conditions to better establish its robustness.

## Supplementary material

10.2196/75516 Multimedia Appendix 1 Decision framework for selecting the calibration parameter µ in Weighted Gain Score calculations according to the evaluation objective (absolute adjustment, relative adjustment, or routine evaluation).

## Abbreviations

WGS: Weighted Gain Score
RLG: Rasch Learning Gain
