# Supplementary material for: Comparing the Weighted Gain Score and a Rasch-Based Approach for Estimating Learning Outcomes in Medical Education: Quantitative Study
Source: JMIR Med Educ. 2026 Jun 16;12:e75516. doi: 10.2196/75516 (PMC13271600; doi:10.2196/75516)
Supplement: Multimedia Appendix 1 [file mededu-v12-e75516-s001.docx]

**Multimedia Appendix 1**


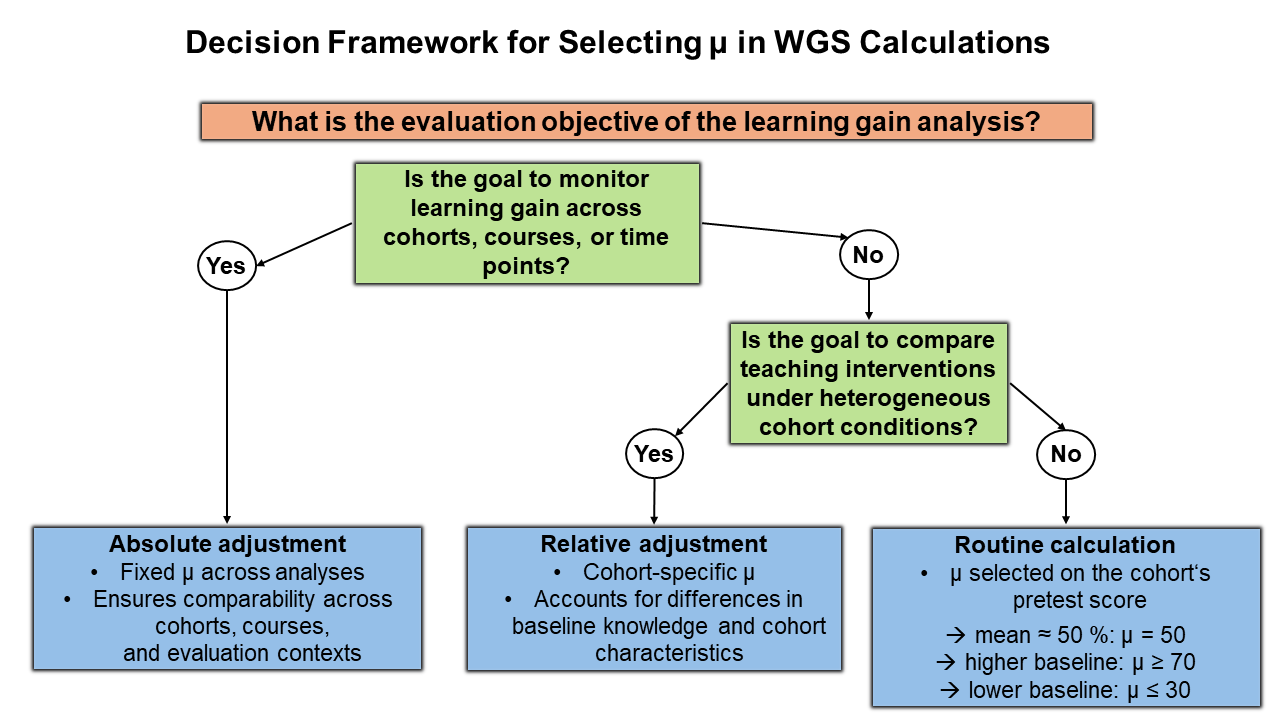


Figure: Decision framework for selecting the calibration parameter µ in WGS calculations depending on the evaluation objective (absolute adjustment, relative adjustment, or routine calculation).
